# Development of a residency program in radiation oncology physics: an inverse planning approach

**DOI:** 10.1120/jacmp.v17i2.5864

**Published:** 2016-03-08

**Authors:** Rao F. H. Khan, Peter B. Dunscombe

**Affiliations:** ^1^ Department of Oncology Department of Physics and Astronomy University of Calgary Calgary AB Canada

**Keywords:** medical physics, training, residency, education, soft skills

## Abstract

Over the last two decades, there has been a concerted effort in North America to organize medical physicists’ clinical training programs along more structured and formal lines. This effort has been prompted by the Commission on Accreditation of Medical Physics Education Programs (CAMPEP) which has now accredited about 90 residency programs. Initially the accreditation focused on standardized and higher quality clinical physics training; the development of rounded professionals who can function at a high level in a multidisciplinary environment was recognized as a priority of a radiation oncology physics residency only lately. In this report, we identify and discuss the implementation of, and the essential components of, a radiation oncology physics residency designed to produce knowledgeable and effective clinical physicists for today's safety‐conscious and collaborative work environment. Our approach is that of inverse planning, by now familiar to all radiation oncology physicists, in which objectives and constraints are identified prior to the design of the program. Our inverse planning objectives not only include those associated with traditional residencies (i.e., clinical physics knowledge and critical clinical skills), but also encompass those other attributes essential for success in a modern radiation therapy clinic. These attributes include formal training in management skills and leadership, teaching and communication skills, and knowledge of error management techniques and patient safety. The constraints in our optimization exercise are associated with the limited duration of a residency and the training resources available. Without compromising the knowledge and skills needed for clinical tasks, we have successfully applied the model to the University of Calgary's two‐year residency program. The program requires 3840 hours of overall commitment from the trainee, of which 7%–10% is spent in obtaining formal training in nontechnical “soft skills”.

PACS number(s): 01.40 Di, 01.40.gb, 87.10‐e

## I. INTRODUCTION

With the recent requirement by the American Board of Radiology that candidates for the certification examination must have completed a CAMPEP accredited residency, there has been a concerted effort across the continent to formalize both the structure of residency programs themselves as well as the standards against which they are assessed. On a similar timescale, there has been increasing emphasis on topics such as ethics and professionalism and patient safety. The AAPM published the Report of Task Group 109: *Code of Ethics for the American Association of Physicists in Medicine* in 2009, and the Report of Task Group 159: *Recommended ethics curriculum for medical physics graduate and residency programs* in 2010. The American Board of Radiology Foundation released its online Ethics and Professionalism Program in 2011.^(1)^ Patient safety and error management have become recurring themes at national and international meetings. Tools for enhancing the safety and quality of radiotherapy within ongoing resource constraints are described in detail in the forthcoming anticipated report of the AAPM's Task Group 100.^(2)^ The challenge for medical physics residencies is how to bring these important aspects of the formation of the medical physicist to life within a residency program as opposed to merely treating them as add‐ons to be incorporated in an ad hoc informal basis. This challenge is all the greater given the paucity of educational materials at a level and orientation appropriate to the professional medical physicist.

Clearly, the primary focus of a medical physics residency program is, and should continue to be, to develop a high level of clinical physics competency in the graduating resident.^(3)^ A parallel statement could be made for medical residencies. However, in recent years the medical profession has started to recognize that, while clinical (physics) competence is a prerequisite for employment in the radiotherapy clinic, it does not, on its own, lead to optimal functioning in the today's health care environment. Twenty years ago the Royal College of Physicians and Surgeons of Canada introduced the CanMeds framework.^(4)^

The CanMEDS Physician Competency Framework describes the knowledge, skills and abilities that specialist physicians need for better patient outcomes. The framework is based on the seven roles that all physicians need to have, to be better doctors: Medical Expert, Communicator, Collaborator, Manager, Health Advocate, Scholar, and Professional.^(4)^

More recently the American Board of Radiology has listed six essential competencies for maintenance of certification for both physicians and physicists: medical knowledge; patient care and procedural skills; interpersonal and communication skills; professionalism; practice‐based learning and improvement; systems‐based practice. While these competencies are currently only expected to be exhibited at the time of recertification, can it be more than a matter of time before the graduating resident will be required to provide evidence of grounding in these competencies? Indeed, these core competencies are now receiving prominence in the AAPM's Report 249, the latest revision of the Guidelines for medical physics residency programs.^(5)^

More recently still the American Society for Radiation Oncology has introduced a Disciplines of Leadership course for members who already have or aspire to leadership roles in Radiation Oncology (http://www.astro.org/Meetings-and-Events/Pilot-Leadership-Course/Course-Program/Course-Curriculum.aspx). Leadership^(6)^ is another soft skill that is being increasingly recognized as worth nurturing for the benefit of the radiation oncology professions and the patients they serve.

While level I evidence of the benefit of highly developed “soft skills” such as communication, collaboration management, and leadership is lacking, it is apparent that many of these skills are becoming recognized expectations of senior health care professionals, such as physicists and physicians.^7^, ^8^

We conclude from a brief review of the recent initiatives by respected educational and certifying organizations that the role of relevant soft skills in the formation of senior health care professionals is assuming, appropriately, greater importance.

Further, we propose that these nontechnical essentials should be formally taught and practiced as part of the curriculum in a professional medical physics residency. As imparting the traditional medical physics competencies has transitioned from an on‐the‐job format to a better defined, structured program, so must these other skills necessary for optimum functioning in today's health care environment. In this paper, we outline an inverse planning approach to the design of a medical physics residency program based on our observations above. We will describe the essential components identified through this approach and how they have been incorporated in the radiation oncology physics residency program at the University of Calgary (UofC).

## II. MATERIALS AND METHODS

At the time of development of the residency program described here, the Medical Physics Department at the cancer center supported the treatment of approximately 2400 new patients per year on nine megavoltage units. Most modern treatment techniques, including low‐ and high‐dose‐rate brachytherapy, IGRT, TBI and motion management, were — and still are — routinely available. There were 11 medical physicists in the Department, all certified by the Canadian College of Physicists in Medicine and all of whom had an academic appointment at some level within the Department of Oncology and/or Department of Physics and Astronomy at the University of Calgary (http://www.ucalgary.ca/rop/). The University of Calgary is a comprehensive university offering the full range of undergraduate and graduate degrees including medicine. In addition to a CAMPEP accredited Residency Program, accredited MSc and PhD programs were also offered.

Our approach to the development of the radiation oncology physics residency program is analogous to inverse planning in radiation therapy. We identify the objectives of the program, while remaining cognizant of the constraints of time and resources during this development exercise.

### A. Objectives

1. To comply with CAMPEP requirements for accreditation of a residency program. The development described here was undertaken in 2007 prior to the introduction of the new CAMPEP standards (http://www.campep.org).
2. To allow appropriate academic recognition, through the UofC, of the efforts of faculty in contributing to resident training.
3. To introduce residents to selected fundamentals of effective functioning and leadership in a complex environment.
4. To formalize an ethics component of the program.
5. To formalize a patient safety/error management component of the program.
6. To promote skill development in teaching.
7. To expose residents to some of the important “soft skills” required in the clinical environment.

### B. Constraints

1. The program had to be completed within the two years of the residency.
2. The program had to be implemented within the interests, resources, and organizational/ academic structures of the cancer center and the UofC.

## III. RESULTS

Below we describe how we have attempted to meet the set objectives.

### A. Objective 1: Compliance with CAMPEP requirements

A sufficient depth of clinical physics knowledge and the development of clinical skills will continue to remain the cornerstone of any medical physics residency. Residents entering this, and most other programs, have already acquired the required basic level of medical physics knowledge through a prior graduate or certificate program — AAPM report 197s.^(9)^

Fortunately, we can use, as the required knowledge base, the plethora of published literature in medical physics in the forms of review articles, task group reports from the AAPM, and other procedure specific documents (http://www.aapm.org/pubs/reports/). This knowledge must be organized into strategically planned curricula and linked to specific objectives. Finally, incorporating regular and rigorous assessment will determine the degree to which an individual attains the required level of knowledge and performance. The major courses and practica listed below systematically improve both knowledge and skill by progressing upward on Miller's pyramid^(10)^ from knowing to doing. [Note: Courses identified by * constituted six of the required eight courses for the post‐PhD Diploma program, described below.)

MDPH 711(/712)* — Clinical Competency 1 (/2): These structured courses extend over the first (/second) year of the residency and consist of rotations through areas of clinical physics, with extensive reading lists, under the supervision of faculty. Rigorous oral evaluations take place throughout these courses.

MDPH 731* — Radiation Oncology Physics Tutorials: This course requires the resident to prepare written answers to 120 preset questions published by the Canadian College of Physicists in Medicine as part of the certification process in Radiation Oncology Physics in Canada. The course is conducted in a tutorial setting, with examinations and evaluations carried out periodically.

MDPH 741 * — Treatment Planning: This course has three components. The first component is the observation of simulation and localization under the supervision of a radiation oncologist. The second component is an in‐depth study of treatment planning physics. Human anatomy and physiology is refreshed by using Web‐based tools (such as free YouTube lectures from UC Berkley website). The final component involves following 10 patients through their entire radiation therapy course, from immobilization to treatment delivery. The resident's progress is evaluated throughout the course with regular written and oral exams.

The graduating resident will be expected to have acquired more extensive hands‐on experience with the technology and techniques of modern radiotherapy than can be gained during the clinical competency courses which provide merely an overview. The acquisition of critical clinical skills is accomplished through several mechanisms. Firstly, all residents take responsibility, under supervision, for routine quality control on accelerators, simulators, and other equipment over the full two years of the residency. After a short introduction period, each resident is assigned a routine clinical task such as quality control of one of the linear accelerators. Every resident contributes to annual quality control. Rotation around clinical tasks is encouraged and expected to maximize the exposure of the resident to the range of equipment and techniques in the clinic. Secondly, two formal numbered and structured University of Calgary project courses are required components of the residency. Lastly four practica are taken over the two summers of the residency.

MDPH $712/722^{*}$. Two to three short term clinical projects are completed during each course extending over the first (/second) year of the program. Projects with immediate clinical relevance, such as a contribution to the commissioning of a new machine, and with clearly defined objectives are offered by the faculty. The projects culminate in written reports, oral presentations, and occasionally a journal publication. The resident's performance is formally evaluated against the objectives established at the commencement of each project.

Clinical experience is augmented by four structured supervised practica, taken over the two summers of the program: two in treatment planning and another two in diagnostic imaging. Table 1 entails the course scheduling and in‐class contact hours for university courses and direct supervision hours for the rest of the modules.

**Table 1:** Components of an inversely planned TBCC radiation oncology physics residency program: ☑ shows a major emphasis, ++ a minor emphasis, and + some emphasis

*Courses/Parameters*	*Clinical Competency‐1*	*Treatment Planning*	*Optimizing Team Dynamics*	*Clinical Projects*	*Instructional Skills Development*	*Ethics & Errors*	*Practica*	*Clinical Competency‐2*	*Radiation Oncol. Phys Tutorials*	*Advanced Leadership & Technical Skills*
Clinical knowledge & Critical skill	☑	☑		☑		☑	☑	☑	☑	*+*
Interpersonal & communication
skills	*++*	*++*	☑	☑	☑	*++*	☑		☑	☑
Decision making & leadership	+	+	*+*	*++*	*++*	+	☑	☑	*+*	☑
Professionalism	+	+	☑	+		☑	☑	☑	*+*	*+*
Class room contact hours/Direct supervision	40 h	80 h	40 h	80 h	30 h	30 h	480 h	40 h	80 h	40 h
Schedule	Sep to Dec (Yr.1)	Sep to Apr (Yr.l)	Sep to Dec (Yr.l)	All years	Sep to Apr (Yr.1)	Jan to Apr (Yr.2)	Apr to Sep (Yr.1 & Yr.2)	Sep to Dec (Yr.2)	Sep to Apr (Yr.2)	Sep to Dec (Yr.2)
Mode of learning	Participatory, discussions with tutors	Participatory, peer discussions, lecturing, Web‐tools	Participatory, team assignments	Individual with supervision	Small and large group	Small group, lecturing	Participatory, discussion	Participatory, discussions with tutors	Participatory, discussions with tutors	Participatory, team assignments
Assessment tools	Participation, assignments, written & oral exams	Participation, assignments, Web‐based MCQ exam, Written oral and competency exam	Group learning, assignments	Project report, presentation	Participation, 360‐degree evaluation, presentation of lesson, peer teaching	Participation, involvement	Participation, presentation	Participation, assignments, written & oral exams	Written assignments	Group learning, assignments

### B. Objective 2: Academic recognition for faculty

There were three reasons for incorporating a post‐PhD Diploma program within the two‐year residency. The first was to formalize recognition of the faculty for their contributions to this advanced level of training and the associated demands on the faculty. For the purposes of academic promotion and career progression, contributions to numbered courses in the University of Calgary calendar carry much more weight than ad hoc informal teaching activities. Secondly, as described below, it was necessary to formalize the relationship between the residency program and the UofC in order to access the heavily oversubscribed Masters in Business Administration courses required to meet Objective 3. Finally, alignment with University standards enforces a degree of rigor on both faculty and trainees in terms of timely completion of the individual course requirements, thorough documentation and evaluations both by, and of, the trainee.

A Diploma program at the University of Calgary requires equivalent of 10 credit courses (10 at 40 hours each or equivalent of student contact time). The courses described under Objective 1 above (and identified by *) constituted six of the required eight courses, with the two remaining being the business courses described below. While incorporation of the post‐PhD Diploma did confer benefits on the residency program, it came with its own constraints such as scheduling courses in synchrony with the University's semester system. It was felt that adequate exposure to treatment planning and the many imaging devices necessary for modern radiotherapy could not be accomplished within the constraints of the Diploma structure. For that reason, two practica were scheduled for each of the two summers of the residency program.

### C. Objective 3: Effective functioning and leadership in a complex organization

Introduction to these management topics is addressed primarily through the two graduate business courses offered through the University of Calgary's Haskayne School of Business Masters in Business Administration program.

HROD 793* — Business Negotiations: The major concepts and theories of negotiation, dynamics of interpersonal and intergroup conflict, and analysis of negotiation strategies and individuals’ styles are discussed, along with simulations and applications from various areas.

HROD 789* — Optimizing Team Dynamics is designed for those who will be leading or working in teams in environments where success depends on effective teamwork and the ability of team members to integrate disparate knowledge bases.

Participants in these courses include leaders from industry and government, as well as from the health care sector. MBA courses in most universities are oversubscribed and hence do not accept “walk‐ins”. Approval of the Post‐PhD, Diploma program followed by negotiation with the Business School led to the allocation of guaranteed slots for our residents.

### D. Objectives 4 and 5: Ethics and Error Management

Systematic treatment of errors and uncertainties have evolved only recently from cross marriage from safety and reliability engineering. However, it has received little attention in the training programs of not just medical physicists, and has been identified as a major shortcoming in allied residencies’ models.^11^, ^12^ A recent survey of the AAPM members recognized importance of formal ethics and professionalism instruction in both graduate and postgraduate training of medical physicists.^(13)^ The residency program described here was developed prior to the availability of the AAPM/ASTRO online course in Ethics and Professionalism^(1)^ and the AAPM Report 109^(14)^: Code of Ethics. There were no readily identifiable educational resources geared towards the medical physics resident and which could be covered without compromising the clinical experience of the resident. The same was true of error management in the radiotherapy environment, which has assumed increasing importance over the last two decades. As these two topics were considered of major importance in a comprehensive preparation for clinical practice, the decision was made to develop one course covering these two topics from scratch.

The course has been described elsewhere.^(15)^ Briefly, there are five 3‐hour ethics modules and seven 3‐hour errors modules. Approximately half the class time is devoted to group exercises in which participants, which included senior graduate students as well as residents, tackled issues involving ethics in research, education, and clinic, and gained familiarity with some of the techniques of error management such as Failure Modes and Effects Analysis. Due to shorter length of the course, it is not run through the university.

### E. Objective 6: Teaching skill development

Residents are required to take an Instructional Skills Workshop through the University of Calgary (http://tlc.ucalgary.ca/teaching/programs/isw). Many of our trainees were sufficiently motivated to pursue the University of Calgary teaching certificate which requires further study, culminating in an assessment of the resident's teaching skills in a classroom setting. Following completion of the Instructional Skills Workshop, the resident is then assigned to teach about 20 to 36 hours of graduate classroom teaching, (http://www.ucalgary.ca/rop/graduate_program), and teaching other professionals.

### F. Objective 7: Soft skills development

There is set of basic skills required for effective functioning in any environment. These include communication, negotiation, leading and participating in meetings, and supervision. A one‐and‐a‐half‐day (12‐hour) course was developed to engage trainees in these topics and included role playing to demonstrate some of the more common pitfalls when practicing these activities.^(16)^ The central theme throughout the scenarios presented was emotional intelligence, which many believe to be a key attribute of effective leaders.^(17)^

**Figure 1:**
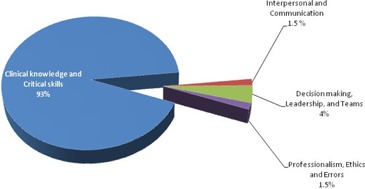
Relative time commitment to several components of UofC radiation oncology physics residency program. The ‘clinical knowledge and critical skills’ sums up to 3560 hr (93% of the overall commitment 3840 hr). The commitment to three ‘soft skills’ is obtained by doubling the in‐class hours (from Table 1) to account for effort involved in taking the course work.

Table 1 and Figure 1 summarize the program structure, including the time commitment to the various components.

The program has included all the components listed above since 2007, with the exception of the “soft skills workshop” which was added in 2012. The presence of structure through the university system provides a framework for formal evaluations and improvements to the training program for most of the components. All components are evaluated by the residents and any comments made are used to modify the program when possible. We conclude from both formal and informal evaluations that the program has been well received by the residents.

## IV. DISCUSSION

Reconfiguring this residency program, whilst maintaining a solid core of clinical competency development, was predicated on several premises. Firstly, faculty who engage in training should be formally recognized for such activities. This is particularly important for junior staff seeking career progression and is best accomplished through university calendar courses that are evaluated according to university procedures.

The clinical physicist may discharge many of her duties alone, such as machine quality control and plan checking. While these core activities are necessary to maintain safety and quality^(18)^ within the clinical program, knowledge and skills not traditionally associated with the medical physicist are necessary if the field is to move forward. The implementation of new, complex technologies is increasingly collaborative, with the roles of the physicist, oncologist, therapist, and dosimetrist constantly evolving. There is little preparation in current training programs for physicists to manage and lead in this constantly changing environment. Incorporation of the two MBA courses and development of the “Street Smarts” program was an attempt to start to address these deficiencies. It is worth remembering that, until CAMPEP and the AAPM started to formalize the structure of residency programs, on‐the‐job training was accepted as an adequate preparation for the clinical physicist. If on‐the‐job clinical training is no longer accepted as appropriate for the professional medical physicist, then other competencies essential for effective high‐level functioning in a complex multidisciplinary environment should also be developed within a formal structure. As already pointed out, there is a lack of educational materials geared towards medical physicists, which makes incorporation of these topics within a residency program challenging.

Next, training programs need to acknowledge ethical behavior and professionalism as fundamental attributes of medical professionals.^(19)^ This recognition has come slowly. Since the program described here was implemented, the AAPM/ASTRO have developed an online ethics course and the AAPM has released Reports 109^(14)^ and 159^(20)^ and some online tools have started to appear on the horizon^(21)^ In its new standards, CAMPEP has included a whole section on these topics. While these developments are promising, there is still a need to bring these topics to life in residency programs by having knowledgeable faculty engage the trainee in discussions and exercises. Unfortunately, again, there is a paucity of educational material oriented to medical physicists on these topics. The same observation is valid in the area of patient safety and error management. While these topics have captured the public's — and our — attention over the last decade, there are few educational resources to support faculty in imparting the necessary knowledge and attitudes to trainees.

Finally, education and training of others, whether it be through in‐services, graduate level courses or resident mentoring, are expected as important activities of the majority of medical physicists. Only 20% of internal medicine residency program formally train their residents in teaching skills;^(22)^ there are no published data specific for the medical physics programs. To expect that we reach our full potential as educators/trainers just through years of experience is unrealistic. Education and training are research fields in their own right. In order that the newly minted medical physicist can make an immediate impact in the educational arena, he should have been exposed to the latest approaches to adult education and be expected to have developed a degree of competence in this area. Providing formal education and training in pedagogical techniques can help the residents in their own learning, knowledge, and skill development.^(23)^

Figure 1 provides a summary of the overall time commitment to the hard core clinical skills and softer components of the UofC training program. Introducing soft skills as formal part of program takes away about 7%–10% of the overall program. Another note of caution regarding the model is a varying degree of effort required in various courses, though they are all three credit hours.

A constraint that was not identified at the start of development of this program was the additional financial burden of enrolling in a university program. This was the price of meeting optimization Objectives 2 and 3. The cost of the post‐PhD Diploma embedded in the residency program is approximately $10,000 over two years, with most of this fee resulting from the two MBA courses ($8000). Applicants to the program have to decide whether or not the additional features of this program justify the additional cost. However, to put this cost in today's education and training context for medical physics, a Certificate program, lasting a year or less, costs between $8000 and $15,000, while the residency component of a Doctorate in Medical Physics Program has similar costs for the resident, however, per year. Providing exposure to residents regarding formal management skills and decision‐making processes during a training program opens new venues where an enthused trainee can hone his/her skills by going through a full professional degree program such as MBA. Moreover, if in future the medical physics discipline needs to incorporate the leadership and management skills as part of a formal degree program, one can extend the residency program to a three‐year MBA/Post‐PhD diploma.

## V. CONCLUSIONS

A program has been developed and implemented which met the tested and tried objectives from medicine and allied residencies^2^, ^6^, ^19^ and identified at the start of the exercise and which operated within the constraints. Although this program was developed prior to the development of formal standards by the CAMPEP, it not only encompasses those standards referring to such issues as ethics and professionalism,^(24)^ patient safety and collaborative working, but it also formally incorporates them into the program, as opposed to an ad hoc approach relying on senior staff transferring their wisdom through occasional contact with the resident.^(12)^ As with any other educational and training program, there is always scope for improvement. However, it is time to acknowledge that skills beyond those in clinical physics are required by the modern medical physicist if her full potential contribution to the treatment of patients is to be realized. Professional organizations, such as the AAPM, could consider developing educational and training packages which will guide and assist faculty of residency programs in delivering soft‐skill components within their own programs.

## COPYRIGHT

This work is licensed under a Creative Commons Attribution 4.0 International License.
